# Total neoadjuvant treatment and PD-1/PD-L1 checkpoint inhibitor in locally advanced rectal cancer

**DOI:** 10.3389/fimmu.2023.1149122

**Published:** 2023-03-24

**Authors:** Weiwei Xiao, Huilong Luo, Ye Yao, Yaqin Wang, Shuang Liu, Rui Sun, Gong Chen

**Affiliations:** ^1^ Department of Radiation Oncology, State Key Laboratory of Oncology in South China, Collaborative Innovation Center for Cancer Medicine, Sun Yat-Sen University Cancer Center, Guangzhou, China; ^2^ Department of Colorectal Surgery, State Key Laboratory of Oncology in South China, Collaborative Innovation Center for Cancer Medicine, Sun Yat-Sen University Cancer Center, Guangzhou, China

**Keywords:** locally advanced rectal cancer, total neoadjuvant treatment, long-course radiotherapy, PD-1/PD-L1, short-course radiotherapy

## Abstract

For local advanced rectal cancer (LARC), total neoadjuvant treatment (TNT) has shown more complete response (CR), reduced risk of distant metastasis (DM) and increase of the sphincter preservation rate. Now it is the one and only recommendation for high-risk group of LARC according to National Comprehensive Cancer Network (NCCN) rectal cancer guideline, while it is also preferentially recommended for low-risk group of LARC. TNT is also beneficial for distant rectal cancer patients who have need for organ preservation. Even though the prognostic value of programmed cell death-ligand 1 (PD-L1) in the neoadjuvant chemoradiotherapy (NACRT) of LARC patients is undetermined yet, the combination of NACRT and programmed cell death-1 (PD-1)/PD-L1 antibodies seem bring new hope for mismatch repair proficient (pMMR)/microsatellite stable (MSS) LARC patients. Accumulating small sample sized studies have shown that combining NACRT with PD-1/PD-L1 antibody yield better short-term outcomes for pMMR/MSS LARC patients than historic data. However, ideal total dose and fractionation of radiotherapy remains one of unresolved issues in this combination setting. Thorough understanding the impact of radiotherapy on the tumor microenvironment and their interaction is needed for in-depth understanding and exquisite design of treatments combination model.

## 1 Introduction

Neoadjuvant chemoradiotherapy (NACRT) or short-course radiotherapy (SCRT) followed by total mesorectal excision plus adjuvant chemotherapy (ACT) was the standard treatment modality for locally advanced rectal cancer (LARC). Despite the improvement in local control with the standard treatment regimen, the rate of distant metastasis (DM) is still as high as 35% (1). Additionally, chemoradiotherapy (CRT) plus total mesorectal excision surgery may result in the impairment of defecation, urinary and sexual function. Thus, perioperative treatment strategies are needed to be further developed to decrease surgical morbidity and improve quality of life in LARC patients.

**Figure 1:**
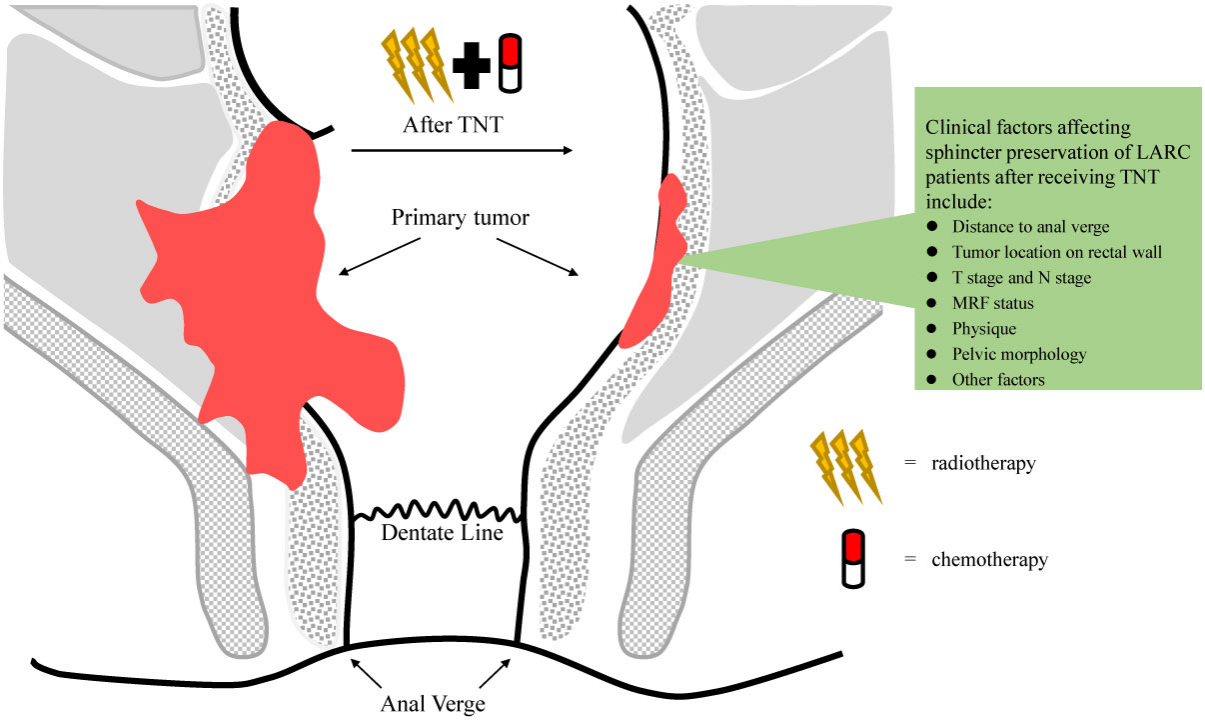
Tumor regression in patients with local advanced rectal cancer (LARC) receiving total neoadjuvant treatment (TNT).

Total neoadjuvant treatment (TNT), a new treatment strategy, shifts all or part of adjuvant chemotherapy to the preoperative phase in the setting of CRT, increases neoadjuvant chemotherapy cycles and prolongs surgical waiting time. The LARC patients who received TNT had better tumor regression and some of them underwent sphincter-preservation operation instead of abdominal pelvic resection (APR). More importantly, there is greater chance that the surgery can be avoided when LARC patients achieve clinical complete remission (cCR) after TNT (2) (**Figure 1**). Moreover, TNT also can contribute to solving the problem of insufficient adjuvant chemotherapy due to surgical complications and consequent poor compliance. It intensifies chemotherapy before total mesorectal excision in order to reduce the risk of DM. In recent years, the administration of immunotherapy combined with TNT or TNT-like treatment has become a hot research topic. As reported by Jing Jin et al, treatment guidelines in the China also recommend TNT as an option for LARC patients (3). Our team also performed a comprehensive meta-analysis to determine the roles of TNT in improving the pathologic complete response (pCR) and survival value compared with standard CRT among LARC patients (4). Major information of clinical trials with published data comparing TNT with standard CRT is summarized in **Table 1** (5–12). In this review, we will discuss the evolution and progress of treatment regimens for LARC patients.

**Table 1:** Randomized clinical trials comparing total neoadjuvant treatment (TNT) with standard chemotherapy treatment (CRT) in LARC.

Study	Author/PI	Publication year	Study ID numbers	Phase	Sample size		Study design	Results
					TNT treatment	Standard treatment		
Maréchal/2011 (5)	Raphaël Maréchal et al.	2011	EudraCT: 2006-006646-34	Phase 2 RCT	28	29	mFOLFOX6 × 2 - CRT [5FU + 50.4 Gy] - TME	ypT0-1N0 rate: 32.1% vs. 34.5% (p= .85)
							vs.	pCR rate: 25% vs. 28% (p= .92)
							CRT [5FU + 50.4 Gy] - TME	
GCR-3 (6)	Carlos Fernandez-Martos et al.	2015	NA	Phase 2 RCT	56	52	Capeox × 4 - CRT [Capeox + 50.4 Gy] - TME	5-year DFS: 62% vs. 64% (p= .85)
							vs.	5-year OS:75% vs. 78% (p= .64)
							CRT [Capeox + 50.4 Gy] - TME - Capeox × 4	5-year cumulative LR: 5% vs. 2% (p= .61)
								5-year cumulative DM: 23% vs. 21% (p= .79)
PRODIGE 23 (7)	Thierry Conroy et al.	2021	NCT01804790	Phase 3 RCT	231	230	mFOLFIRINOX × 6 - CRT [50.4Gy/25F + Cape] - TME - mFOLFOX6 × 6/Cape × 4	pCR: 28% vs. 12% (p<.0001)
			PRODIGE 23 - UCGI 23				vs.	3-year DFS: 76% vs. 69% (HR=0.69, 95%CI: 0.49-0.97; p= .034)
							CRT [50.4Gy/25F + Cape] – TME - mFOLFOX6 × 12/Cape × 8	3-year OS: 91% vs. 88% (HR=0.65, 95%CI: 0.40-1.05; p= .0773)
								3-year MFS: 79% vs. 72% (HR=0.64, 95%CI: 0.44-0.93; p= .017)
POLISH II (8)	Krzysztof Bujko et al.	2016	NCT00833131	Phase 3 RCT	261	254	5 × 5Gy - FOLFOX4 × 3 -TME	pCR: 16% vs. 12% (p= .17)
			PGBRJG0109				vs.	Median OS: 89 months vs. 81 months
							CRT [5FU + LV + OXA + 50.4 Gy] - TME	8-year DFS: 43% vs. 41%
WAIT (9)	James Moore et al.	2017	ACTRN12611000339954	Phase 2 RCT	25	24	CRT [5FU + 50.4 Gy] -5FU + LV × 3 -TME	pCR rate: 16% vs. 25% (p= .49)
							vs.	cCR rate: 12% vs. 8.3% (p= 1.0)
							CRT [5FU + 50.4 Gy] - TME	
KCSG CO 14-03 (10)	Ji Yeon Baek et al.	2018	NCT01952951	Phase 2 RCT	53	55	CRT [Cape/5FU + 50.4 Gy] -Capeox × 2 -TME	pCR: 13.6% vs.5.8% (p= .167)
			KCSG CO14-03				vs.	Downstage rate: 36.4% vs. 21.2% (p= .077)
							CRT [Cape/5FU + 50.4 Gy] - TME	MPR rate: 29.5% vs. 19.2% (p= .167) Mean NAR: 15.66 vs. 20.59 (mean difference= 4.93, 95% CI: 0.20-10.06; p= .06)
STELLAR (11)	Jing Jin et al.	2019	NCT02533271	Phase 3 RCT	302	297	5 × 5Gy - Capeox × 4 - TME ± Capeox × 2 vs CRT [Cape + 25 × 2Gy] - TME ± Capeox × 6	pCR+cCR: 21.8% vs. 12.3% (p= .002)
			XT2015-03				vs.	3-year DFS: 64.5% vs. 62.3% (HR=0.88, 95% CI: not applicable to 1.11; Noninferiority test p<.001)
			CH-GI-090				5 × 5Gy - Capeox × 4 - TME ± Capeox × 2 vs CRT [Cape + 25 × 2Gy] - TME ± Capeox × 6	3-year OS: 86.5% vs. 75.1% (HR=0.67, 95%CI: 0.46-0.97; p= .033)
								3-year MFS: 77.1% vs. 75.3% (HR=0.88, 95%CI: 0.63-1.24; p= .475)
RAPIDO (12)	Geke A P Hospers et al.	2020	NCT01558921	Phase 3 RCT	462	450	5 × 5Gy - Capeox × 6/FOLFOX4 × 9-TME	pCR: 28% vs. 14% (p<.0001)
			NL36315.042.11				vs.	3-year disease-related treatment failure: 23·7% vs. 30·4% (HR=0.75, 95%CI: 0.60-0.95; p= .019)
			2010-023957-12 (EudraCT Number)				CRT [Cape + 28×1.8Gy/25 × 2Gy] - TME - Capeox × 8/FOLFOX4 × 12	3-year OS: 89.1% vs. 88.8% (HR=0.82, 95%CI: 0.67-1.25; p= .59)
								3-year DM: 20.0% vs. 26.8% (HR=0.69, 95%CI: 0.54-0.90; p= .0048)

RCT, randomed clinical trial; CRT, chemoradiotherapy; TME, total mesorectal excision; mFOLFOX, modify oxaliplatin + leucovorin + 5-fluorouracil; Capeox, capecitabine + oxaliplatin; mFOLFIRINOX, modify oxaliplatin + irinotecan + calcium folinate + 5-fluorouracil; Cape, capecitabin; 5FU, 5-fluorouracil; AF, ; LV, leucovorin; OXA, oxaliplatin; FOLFOX, oxaliplatin + calcium folinate + 5-fluorouracil; cCR, clinical complete response; pCR, pathologic complete response; DFS, disease free survival; OS, overall survival; LR, local relapse; DM, distant metastases; MFS, distant metastasis-free survival; MPR, major pathological response; NAR, neoadjuvant rectal score; HR, Hazard Ratio.

## 2 When should we select TNT strategy?

According to recent National Comprehensive Cancer Network (NCCN) rectal cancer guideline, risk classification was crucial in neoadjuvant treatment decisions for LARC patients. LARC patients were stratified into the low risk (T3Nany with clear circumferential resection margin (CRM) or T1-2N1-2) and high risk (T3Nany with involved or threatened CRM, T4Nany or locally unresectable or medically inoperable) groups based on 2021 NCCN guidelines (version 1) (13). For the high-risk group, patients are suggested strongly to perform TNT rather than NACRT, and the recommendation continues to date (14). Several TNT treatment regimens, including LCRT or SCRT followed by sequential chemotherapy and induction chemotherapy plus LCRT or SCRT, are available to high-risk LARC patients. RAPIDO trial, a phase 3 randomized controlled trial (RCT), with 3-year disease-related treatment failure as primary endpoint, enrolled high-risk LARC patients, such as cT4, cN2, extramural vascular invasion, involved mesorectal fascia, and enlarged lateral lymph nodes (12). The results showed that rate of disease-related treatment failure decreased significantly in TNT group compared with standard NACRT (Hazard Ratio (HR) = 0.75, 95% Confidence interval (CI): 0.60–0.95, p= .019). And there was also significant difference in distant metastasis-free survival (DMFS) (HR = 0.69, 95% CI: 0.54 to 0.90, p = .005) between TNT group and CRT group. However, no significant difference in overall survival (OS) was observed between the two groups (HR = 0.92, 95% CI: 0.67–1.25, p= .59). Exceptionally patients with lateral lymph node (LLN) metastasis may benefit from NACRT strategy although the difference was not statistically significant shown in the forest map. This suggested that, for patient with positive LLN, TNT treatment led to the protracted surgical waiting time or SCRT may also bring severe fibrosis than long course radiotherapy (LCRT), which ultimately had a negative impact on survival outcomes. Nevertheless, clinical practice guidelines from both Chinese Society of Clinical Oncology (CSCO) and European Society for Medical Oncology (ESMO) also recommend TNT as preferred treatment approach for high-risk LARC patients (15, 16).

For low-risk LARC patients, both standard NACRT and TNT were suggested by the 2021 NCCN guideline (version 1) (13). But in the 2022 NCCN guideline (version 3), TNT was preferred for low-risk LARC patients although standard NACRT is still a choice (14). The updates of treatment paradigm in recent NCCN guidelines for low-risk LARC patients were based on the results of several RCTs (7, 11). For example, PRODIGE23 trial, enrolled all LARC patients regardless of risk classification, showed higher 3-year disease-free survival (DFS) rate (HR = 0.69, 95% CI: 0.49–0.97, p= .034) and 3-year DMFS rate (HR = 0.64, 95% CI: 0.44–0.93, p= .017) in TNT arm compared with standard NACRT arm (7). The difference of 3-year OS between TNT and standard NACRT arms was not statistically significant (HR = 0.65, 95% CI: 0.40–1.05, p= .0773), most likely because the sample size was relatively small. Unfortunately, subgroup analysis of the PRODIGE23 study based on the tumor stage and risk classification hasn’t been reported yet. Hence, it remained unclear whether low-risk LARC patients stood to benefit the most from TNT. Similarly, in another prospective and phase III STELLAR trial, patients with distal or middle-third, cT3-4 Nany rectal cancer were randomly assigned to SCRT plus four cycles of CAPOX (TNT-like arm) or standard NACRT arm (11). The results showed that TNT-like arm achieved a higher three-year OS rate compared to CRT arm (86.5% vs. 75.1%, p = .033). At 3 years, the cumulative probability of DFS was 64.5% in the TNT-like arm compared with 62.3% in CRT arm (HR = 0.883), even though its design was a non-inferior study.

In a retrospective study presented at ASCO GI 2019, William Chapman et al. compared the clinical outcomes of SCRT in the setting of TNT (SC-TNT) to standard CRT for LARC patients (17). The results suggested that PROSPECT-eligible patients had better DFS in SC-TNT strategy compared with standard NACRT strategy although the difference was not statistically significant potentially due to the small sample size (17, 18). The randomized PSSR study, which enrolled low-risk LARC patients with negative magnetic resonance imaging (MRI)-predicted CRM, also compared direct surgery plus selective CRT with standard NACRT followed by surgery and adjuvant chemotherapy (19). Significant difference in the 3-year cumulative incidence of DFS between the upfront surgery group (81.1%, 95%CI: 77.3%-84.9%) and NACRT group (86.6%, 95%CI: 82.7%-90.5%) was reported at 2022 ASCO meeting (HR = 2.02, 95%CI: 1.01-4.06, p= .048). And the difference of rates for 3-year DFS was 5.4% (95%CI: 5.3%-5.6%), which failed to meet its predetermined criterial of noninferiority. It suggested that NACRT was essential even for low-risk LARC patients with negative MRI-predicted CRM. For LARC with uninvolved mesorectal fascia (MRF), neoadjuvant chemotherapy with CAPOX may be another effective treatment strategy as it yielded similar pCR (11.0% vs. 13.8%, p= .33) and downstaging rates (40.8% vs. 45.6%, p= .27) compared to NACRT in CONVERT trial (20). Another prospective, phase II/III randomized PROSPECT study, including any cT2 cN1 or cT3 cN1-2 rectal cancer patients, now is in progress to determine whether neoadjuvant chemotherapy (FOLFOX or CAPOX) could be used as an alternative to NACRT (18). It is urgent to identify the optimal treatment strategy for low-risk LARC patients.

Owing to special anatomical location and functions of rectum, the distance of the tumor from the anal verge is one of the key factors which should be considered when radiation oncologists choose neoadjuvant treatment options for patients. For the rectal cancer patients who desire a sphincter-preserving procedure, TNT strategy downstages tumor and increases cCR probability so that patients are more likely to have sphincter-preserving operation or even avoid surgery to receive watch and wait strategy. This treatment perspective was recommended by 2017 ESMO, 2020 American Society for Radiotherapy and Oncology (ASTRO) and CSCO rectal cancer guidelines (15, 21, 22). Therefore, TNT should be suggested for distal LARC patients, even those with early-stage tumors. PKUCH-R01 trial enrolled patients with mid-low cT2 or early cT3 rectal cancer to receive LCRT followed by 4 cycles of induction CAPOX (23). Of the 64 patients, cCR was achieved in 31 patients (48.4%) and 41 patients (64.1%) received sphincter-preserving surgery. We now are conducting a clinical trial (TESS study, NCT03840239) to determine the minimum number of cycles of chemotherapy in the setting of TNT that can increase the cCR rates of low rectal cancer (24). In the TESS study, patients with low LARC received 2 cycles of CAPOX performed before, during and after LCRT, followed by total mesorectal excision and 2 cycles of adjuvant chemotherapy. This trial has completed enrollment, and the results will be reported soon.

## 3 When should we choose SCRT or LCRT?

The option of neoadjuvant SCRT versus LCRT remains controversial for LARC patients. The differences between two radiation fractionation schemes can be compared under three main aspects as follows: local control rates, toxicity, and the impacts on tumor microenvironment (TME) when combined with immunotherapy.

There were three clinical trials (Polish I, TROG01.04 and Stockholm III) comparing the local recurrence of SCRT followed by immediate surgery with LCRT followed by surgery (25–27). And other three RCTs (Polish II, RAPIDO and STELLAR trials) focused mainly on the comparison of SCRT followed by chemotherapy and subsequent surgery with LCRT followed by surgery (8, 11, 12). In the six RCTs, the local recurrence rates were not significantly different between SCRT and LCRT groups although biologically effective dose (BED) value calculated according to a linear quadratic (LQ) model of SCRT is lower than LCRT. It is unclear whether the similar short-term outcomes between the two groups were related to the underestimated BED values of SCRT calculated by simple LQ model, or diversity of radiosensitivity of rectal tumor.

In terms of therapeutic toxicity, RAPIDO trial showed a higher incidence of acute gastrointestinal toxicity in the SCRT-TNT group compared to the NACRT group (28). Diarrhea was the most common adverse event in both groups. It may be mainly associated with severe intestinal edema caused by hypofractionated radiotherapy. In addition, both 6-months waiting times from the end of radiotherapy to surgery and hypofractionated radiotherapy can lead to intestinal fibrosis, which has an adverse impact on surgical procedures. Intraoperative blood loss was more in the short-course arm compared to the long-course arm (300ml vs 250ml, p= .007). The intact of mesorectal plain as assessed by surgeon were also worse in SCRT-TNT arm compared to NACRT arm (78% vs 85%, p= .032).

## 4 Prognostic value of PD-L1 in LARC after NACRT

Programmed cell death-ligand 1 (PD-L1) pathway mediates immune exhaustion and is a potent target for anticancer immunotherapy.

Lianzhou Yang et al. performed a meta-analysis and found PD-L1 overexpression was relevant to inferior tumor stage (OR= 0.57), vascular invasion-negativity (Odds Ratio (OR)= 0.75), shorter OS (HR= 1.47) and shorter recurrence-free survival (RFS)/DFS (HR= 1.47). But the expression of PD-L1 is not related to age, sex, tumor location, tumor differentiation, pathological T (pT) stage, pathological N (pN) stage, or microsatellite instability (MSI)/mismatch repair (MMR) status (29).

Peter G Alexander et al. focused on the prognostic value of PD-L1 in colorectal cancer receiving anti-PD-1 therapy and published a meta-analysis. They found that programmed cell death-1 (PD-1) on immune cells (iPD-L1) was associated with favorable prognosis, but PD-L1 expression on tumour cells (tPD-L1) has inconsistent outcomes and failed to perform as a useful biomarker (30).

About LARC patients receiving NACRT, accumulating studies reported that PD-L1 expression and T-cell infiltration would increase after NACRT (31–33), however, the association of PD-L1 expression level with tumor response and survival outcomes of LARC after NACRT is not determined yet.

Hecht et al. studied 103 pre-RCT biopsies and 159 post-RCT surgical specimens, and found that low PD-L1 expression in cancer and immune cells (32). Still, both PD-L1 expression in pre-CRT samples and in the invasive front of post-CRT samples were independent positive prognostic markers for OS. Ogura A et al. studied immunostainings of PD-L1 and CD8 in 287 LARC patients (33). tPD-L1 and stromal iPD-L1 expression were evaluated before and after CRT in 287 patients. High iPD-L1 expression significantly increased from 31.7% before CRT to 49.2% after CRT and the increase of iPD-L1 expression was only observed in patients with tumor regression grades 1 and 2. High tCD8+ cell density before CRT was associated with better DFS, but its improved effect on DFS could be only observed in patients with high iPD-L1 expression. Hyungwoo Cho et al. evaluated dynamic changes of TME in patients enrolled in ADORE study and found that high delta values of CD3+ T cells and PD-L1+ lymphocytes after CRT were associated with good DFS, while that of CD4+FoxP3+ regulatory T cells was associated with poor DFS (34).

The above four studies include patients treated with NACRT, while translation study of Voltage clinical trial, which prospectively treated LARC patients with NACRT followed by 5 cycles of Nivolumab, showed that high PD-L1 expression is correlated with higher ratio of pCR (35).

On the contrary, Saigusa et al. reported immunohistochemistry analysis results in 90 LARC patients underwent NACRT (36). Patients with higher PD-L1 expression was significantly associated with more vascular invasion, poor RFS and poor OS. Infiltrating CD8+ T cells in patients with high PD-L1 expression were significantly less than in patients with low PD-L1 expression. Shao L et al. studied the 68 rectal cancer patients treated with neoadjuvant SCRT or standard NACRT and found that tPD-L1 expression is significantly correlated with poor local relapse-free survival (LRFS) (37). Lim YJ et al. performed a paired analysis using pre-CRT and post- CRT tumor tissues of 123 rectal cancer patients undergoing NACRT. Sustained higher expression of PD-L1 at pre- and post-CRT (high-to-high) was associated with less increase in the density of CD8+ tumor infiltrating lymphocytes (TILs) (38). Two subgroups with high baseline PD-L1 expression level, the high-to-low and high-to-high alterations, showed worse OS (HR=8.34 and 11.03, respectively), with the highest mortality risk observed in the high-to-high group. Hiroyuki Takahashi et al. reported study results of 109 NACRT-treated LARC cases (39). They revealed that membranous tPD-L1 was only associated with mismatch repair deficient (dMMR), but not other clinical characteristics. In contrast, iPD-L1 expression were significantly correlated with tumor vessel invasion, nuclear β-catenin-positive tumor budding cancer stem cell (CSC)-like features, and poorer OS.

What’s more, Jomrich G et al. and Richter I et al. reported no PD-L1 expression was noticed rectal cancer tissue before and after NACRT, let alone its prognostic value (40).

In summary, whether PD-L1 expression has also a predictive value for LARC after NACRT is yet unclear. At least, we need to use standardized method to score its expression and use common thresholds enabling comparison between studies and further analysis.

Moying Li et al. summarized biomarkers and tumor models predicting response to NACRT in rectal cancer, which also include TME factors, cytokines and chemokines (41). Consistently, high intratumoral CD8+ and CD4+ lymphocyte infiltration exhibits better survival and better response to NACRT (31, 42–46). And the level of FOXP3+ Treg negatively correlates with responsiveness to NACRT (47, 48). In contrast, there are relatively few reports on the association of the proportion of tumour-associated macrophages (TAM), dentritic cells (DCs) or B cells in TME with tumor response to NACRT.

Liwen Qian et al. established a model based on 15 immune-related genes, which was associated with response to neoadjuvant CRT (49). The 15 immune-related genes were found to be enriched in inflammation pathways through the Gene Set Enrichment Analysis (GSEA). They also showed that CD4 naive T cells, T exhaustion (Tex) cells and T helper 1 (Th1) cells are significantly more while T follicular helper (Tfh) cells are significantly less in responder group than non-responder group.

In another research, patients with MSS tumors were separated into three groups: IG1, IG2 and IG3 (50). Interestingly, IG3 displays features of immunologically hot tumors (immune cell infiltration and elevated immune checkpoint expression) compared with IG1 and IG2. In addition, the study has demonstrated good response to NACRT and prolonged DFS in IG3. But due to the small sample size of IG3, the reproducibility of results remains to be further investigated.

## 5 Adding PD-1/PD-L1 checkpoint inhibitor

The interaction of immune and radiotherapy has been a major focus of research lately. Data on NACRT combined with PD-1 or PD-L1 inhibitors in mismatch repair proficient (pMMR)/microsatellite stable (MSS) rectal cancer patients has been gradually disclosed. A team from Cancer Hospital, Medical Center of Fudan University, led by Fan Xia, has already summarized the available data in detail (51). Detailed information of each trial combining NACRT with PD-1/PD-L1 antibodies for LARC is provided in **Table 2** (35, 52–62).

**Table 2:** Clinical trials of neoadjuvant chemoradiotherapy combined with PD-1/PD-L1 antibodies for LARC.

Study	Author/PI	Initiation Year	Country	Study ID Numbers	Phase	Sample Size	Characteristics	Study Design	Duration of Neoadjuvant therapy (Weeks)	Results
Voltage-A (35)	Takayuki Yoshino et al.	2016	Japan	NCT02948348	Phase 2	39	cT3-4N0-2M0; 23% Stage III; MSS	CRT [50.4Gy + Cape] - Nivolumab × 5 - TME	17	pCR rate: 30% (11/37)
NSABP FR-2 (52)	Thomas J. George et al.	2018	American	NCT03102047	Phase 2	45	Stage II/III; MSS	CRT [n.i.] - Durvalumab × 4 - TME	14	mNAR: 12.03 (p= .06 one-sided)
										pCR rate: 22.2%
										cCR rate: 31.1%
										sphincter preservation rate: 71.4%
PANDORA (53)	Stefano Tamberi et al.	2020	Italy	NCT04083365	Phase 2	55	cT3-4cN+M0	CRT [Cape + 50.4Gy] - Durvalumab × 3 – TME	17	pCR rate: 32.7% (18/55)
Averectal (54)	Ali Shamseddine et al.	2018	Belgium	NCT03503630	Phase 2	44	stage II/III	5 × 5Gy - mFOLFOX × 6 + Ave × 6 - TME	13	pCR rate: 37.5% (15/40)
										MPR: 67.5% (27/40)
Wuhan (55)	Tao Zhang et al.	2019	China	NCT04231552	Phase 2	30	T3-4N0M0 or T1-4N+M0 (86.7% stage III)	5 × 5Gy - Capeox × 2 + Camrelizumab × 2 - TME	8	pCR rate (pMMR): 46.2% (12/26)
										pCR rate(dMMR): 100% (1/1)
AVANA (56)	Lisa Salvatore et al.	2019	Italy	NCT03854799	Phase 2	101	cT4/high risk cT3/cN+(93% stage III)	CRT [Cape + 50.4Gy] + Avelumab × 6 - TME	12	pCR rate: 23% (22/96)
										MPR: 61.5% (59/96)
R-IMMUNE (57)	Javier Carrasco et al.	2017	Belgium	NCT03127007	Phase 2	25	stage II/III (92% stage III)	CRT [5-FU + 50Gy] + Atezolizumab × 4 - TME	15	pCR rate: 24% (6/25)
Changhai hospital (58)	Wei Zhang et al.	2022	China	NCT05215379	Phase 2	23	T1-3aN0-1M0; pMMR/MSS; ultra-low	CRT [50Gy] + Sintilimab × 2 - Cape/Capeox × 6 + Sintilimab × 2 - TME	23	cCR rate: 43.5% (10/23)
										ncCR rate: 26.1% (6/23)
										sphincter preservation rate: 95.5% (21/22)
										CR (pCR + cCR) rate: 52.2% (pCR: 2/10; cCR: 10/23)
Beijing Friendship (59)	Zhongtao Zhang et al.	2022	China	NCT04911517	Phase 2	20	cT3N0M0; cT1-3N1-2M0; <= 10 cm from anal verge; pMMR/non-MSI-H	CRT [Cape + 50Gy] + Tislelizumab × 2 - Cape × 1 + Tislelizumab × 1 – TME	9	pCR rate: 58.7% (7/12)
										NAR: 7.18
NRG-GI002 (60, 61)	Thomas J. George et al.	2016	American	NCT02921256	Phase 2 RCT	Experimental arm: 90	distal location (<= 5 cm from anal verge, any N)/bulky (any cT4 or tumor within 3 mm of mesorectal fascia)/high risk for metastatic disease (cN2)/not a SSS candiate	Experimental arm: FOLFOX × 8 - CRT [Cape + 50.4Gy] + Pembrolizumab × 6 - TME vs FOLFOX × 8 - CRT [Cape + 50.4Gy] - TME	34	mNAR: 11.53 vs. 14.08 (p =.26)
										pCR rate: 31.9% (22/69) vs. 29.4% (20/68) (p= .75)
										cCR rate: 13.9% (11/79) vs. 13.6% (11/81) (p= .95)
						Control arm: 95		Control arm:FOLFOX × 8 - CRT [Cape + 50.4Gy] - TME vs FOLFOX × 8 - CRT [Cape + 50.4Gy] - TME		3-year DFS: 64% vs. 64% (HR=0.95, 95%CI= 0.58-1.55; p= .82)
										3-year OS: 95% vs. 87% (HR=0.35, 95%CI= 0.12-1.00; p= .04)
PKUCH-04 (62)	Zhongwu Li et al.	2020	China	NCT04340401	Phase 2	25	LARC (76% N2, 56% MRF+, 80% EMVI+); pMMR/MSS	Capeox × 3 + Camrelizumab × 3 - CRT [Cape + 50.4Gy] - Capeox × 2 - TME	20	pCR rate: 33.3% (7/21)
										cCR or ncCR rate: 16% (4/25)

RCT, randomed clinical trial; MRF, mesorectal fascia; EMVI, MRI-extramural vascular invasion; pMMR, mismatch repair-proficient; MSS, microsatellite stable; LARC, local advanced rectal cancer; MSI-H, microsatellite instability; SSS, sphincter-sparing surgery; CRT, chemoradiotherapy; TME, total mesorectal excision; mFOLFOX, modify oxaliplatin + leucovorin + 5-fluorouracil; Capeox, capecitabine + oxaliplatin; mFOLFIRINOX, modify oxaliplatin + irinotecan + calcium folinate + 5-fluorouracil; Cape, capecitabin; 5FU, 5-fluorouracil; LV, leucovorin; OXA, oxaliplatin; FOLFOX, oxaliplatin + calcium folinate + 5-fluorouracil; cCR, clinical complete response; pCR, pathologic complete response; DFS, disease free survival; OS, overall survival; MPR, major pathological response; NAR, neoadjuvant rectal score; HR, Hazard Ratio; n.i., no information.

Up to date, the NRG-GI002 trial is the only phase II RCT in which patients with LARC were randomized (1:1) to neoadjuvant FOLFOX for 4 months and then underwent chemoradiotherapy (capecitabine with 50.4 Gy) with or without intravenous pembrolizumab (60). Median neoadjuvant rectal scores (NAR) were 11.53 (95%CI: 8.5-14.6) and 14.08 (95% CI: 10.7-17.4) in the TNT arm and TNT combined with pembrolizumab arm, respectively (p= .26). But the results of other single-arm studies look very encouraging. Although most of patients with high-risk rectal cancer were enrolled in two phase II trials (Wuhan study and Averectal study) in the setting of SCRT with immunotherapy, the pCR rates still approximated 50% (54, 55). For another two LCRT-based studies (Changhai Hospital study and Beijing Friendship Hospital study) enrolled early-stage patients, the pCR rates were above 50% (58, 59).

In LARC, despite it is unclear whether PD-L1 promotes resistance to neoadjuvant chemoradiation, the progress resulting from clinical studies is still quite active, expecting for more data coming.

It seems better antitumor effects could be achieved in both SCRT and LCRT when in combination with immunotherapy. However, Basic questions have not been answered: what dose-fractionation pattern is best? It is necessary to understand the interactions of different radiation fractionation schemes with TME.

TME is composed of stromal cells, immune cells, and molecular components (extracellular matrix, cytokines, and chemokines). The cells in TME vary in radiosensitivity (63). Therefore, different fractionation schemes result in distinct immunological changes within the tumor microenvironment.

A previous review discussed the radiosensitivity of various cells in TME and the change of TME after different dose of irradiation in exquisite detail (64). Proliferating tumor cells is most radiosensitive than stromal cells. Low dose radiation (LDR) promotes apoptosis of tumor cells while high dose radiation (HDR) trigger necrosis, which is characterized by the loss of membrane integrity and the release of damage associated molecular patterns (DAMPs) that contribute to initiate immune responses (64, 65). Additionally, endothelial cell (EC) is resistant to doses up to 10 Gy. Therefore, EC can survival after LDR (<2 Gy). And daily irradiation with a 2 Gy dose can stimulate the process of angiogenesis and vascular permeability to improve tumor hypoxia. Intermediate dose radiation (IDR) can promote the delivery of chemotherapeutics to tumor cells by increasing vascular normalization in tumors, thereby improving the potent of the anti-tumor drug. On the contrary, HDR (>10 Gy) damages vascular EC to increases intratumoural hypoxia, and ultimately, renders cancer cells more resistant to radiotherapy. Within immune cells, DCs are the most radioresistant immune cells. HDR and IDR can trigger anti-tumor response by promoting antigen presentation and activation of DCs while LDR is unable to change the phenotype of DCs. LDR increase the amount of immune-suppressive cells and the production of cytokines, such as M2 macrophages, Myeloid-derived suppressor cells (MDSCs) and transforming growth factor-β (TGF-β) and a high dose of irradiation decreases these immunosuppressive cells and molecules. But high doses per fraction > 10 Gy promotes a hypoxic tumor microenvironment which in turn further aggravates immunosuppression.

Immune system plays a diverse role in different fractionation schemes. In the absent of immune system, 3×8 Gy yielded best tumor control among three different fractionation protocols with similar BED including a single dose of 16.4 Gy, 24 Gy in 3 fraction and 36 Gy in 2 fraction in animal experiments (66). When in immunocompetent murine tumor model, Radiotherapy, whatever the regimen used, improved the tumor-inhibitory effects. Among them, both 18×2 Gy and 3×8 Gy significantly delayed tumor growth. And the study also performed dynamic monitoring of various immune cell subclasses after different fractionation schemes. Conventional fractional radiotherapy (18×2 Gy) increased the number of immunosuppressive cells (such as Myeloid cells, MDSCs and TAM2), while the hypofractionated radiotherapy (1×16.4 Gy or 3×8 Gy) increase the number of immunostimulatory cells (CD8+ and CD4+ T cells). Low-dose radiation also activate anti-tumor immune response by triggering cGAS-STING pathway and type-I interferon pathway. And high-dose radiation promotes the activation and differentiation of T cells and the production of interferon gamma. The selection of immune checkpoint inhibitors is another critical factor to consider when given with different fractionation schemes. The combination of RT (3×8 Gy) and anti-TIGIT and anti-PD-L1 achieve the better CR rate (9/10) compared with all other treatment groups, and the 18×2 Gy is also effective in combination with anti-PD-L1 (CR rate: 8/12).

Tumors with an abundance of infiltrating T-cells appear to be most likely to respond to immune checkpoint inhibitors (ICIs), whereas tumors with an abundance of immunosuppressive myeloid cells and few infiltrating T-cells fail to exhibit a durable response (67). It is necessary to choose optimal fractionation schemes to remodel the TME and enhance efficacy of ICIs. Jessica et al. proposed that different immunotherapeutic strategies should be adopted against different consensus molecular subtypes (CMS) (68). Among all phase III trials for cancer, 78 (3%) investigate a combination of radiotherapy with immunotherapy in one study conducted in 2020. Immunotherapy drugs includes checkpoint inhibitors, cytokines, cell therapy, vaccines, and other targeted immune drugs. These results suggested that with the increasing number of cancer treatment approaches, we need to select the optimal combination of radiation fractionation schemes and immunotherapeutic agent based on the characteristics of tumor. Additional studies are needed to investigate which patients are suitable for SCRT or LCRT.

## 6 Summary

In summary, TNT has become the standard of care for neoadjuvant treatment of rectal cancer, replacing conventional CRT. It can achieve more cCR and reduce the risk of distant metastasis of high-risk patients. The standing role of TNT in high-risk LARC patients has already been established and gained popular usage in clinic. While the value of TNT in low-risk LARC patients is also been praised even though evidence is still accumulating, future studies are still important to gain more evidence on the possible benefits of TNT in low-risk patients. With the discovery of immune activating responses after radiotherapy, there is growing interest in combining NACRT with immune checkpoints to enhance treatment response. Total dose, fractionation, dose distribution and timing of radiotherapy are key variables in determining the effects of radiotherapy on the immune system. In the era of immunotherapy, radiotherapy plays a greater role in clinical treatment of rectal cancer. we are confident that RT combined with immunotherapy will shift the paradigm of treatment strategy of some pMMR/MSS LARC patients.

## Author contributions

GC and WX selected the topic. WX and HL reviewed all published articles and posters, and wrote the manuscript. YY and YW provided some revising suggestions on the Discussion section. YY, SL, and RS revised the manuscript. All authors contributed to the article and approved the submitted version.
